# Quantitative Edge Analysis Can Differentiate Pancreatic Carcinoma from Normal Pancreatic Parenchyma

**DOI:** 10.3390/diagnostics14151681

**Published:** 2024-08-02

**Authors:** Maria Chiara Ambrosetti, Alberto Ambrosetti, Matilde Bariani, Giuseppe Malleo, Giancarlo Mansueto, Giulia A. Zamboni

**Affiliations:** 1 Radiology Unit, Department of Pathology and Diagnostics, Azienda Ospedaliera Universitaria Integrata Verona, 37134 Verona, Italy; matilde.bariani@aovr.veneto.it; 2 Department of Physics and Astronomy “Galileo Galilei”, University of Padova, 35121 Padova, Italy; alberto.ambrosetti@unipd.it; 3 Department of General and Pancreatic Surgery, The Pancreas Institute, University of Verona Hospital Trust, 37134 Verona, Italy; giuseppe.malleo@aovr.veneto.it; 4 Institute of Radiology, Department of Diagnostics and Public Health, Policlinico GB Rossi, University of Verona, 37134 Verona, Italy; giancarlo.mansueto@univr.it (G.M.); giulia.zamboni@univr.it (G.A.Z.)

**Keywords:** computer aided diagnoses, pancreatic adenocarcinoma, glandular surface nodularity

## Abstract

This study aimed to introduce specific image feature analysis, focusing on pancreatic margins, and to provide a quantitative measure of edge irregularity, evidencing correlations with the presence/absence of pancreatic adenocarcinoma. We selected 50 patients (36 men, 14 women; mean age 63.7 years) who underwent Multi-detector computed tomography (MDCT) for the staging of pancreatic adenocarcinoma of the tail of the pancreas. Computer-assisted quantitative edge analysis was performed on the border fragments in MDCT images of neoplastic and healthy glandular parenchyma, from which we obtained the root mean square deviation SD of the actual border from the average boundary line. The SD values relative to healthy and neoplastic borders were compared using a paired t-test. A significant SD difference was observed between healthy and neoplastic borders. A threshold SD value was also found, enabling the differentiation of adenocarcinoma with 96% specificity and sensitivity. We introduced a quantitative measure of boundary irregularity, which correlates with the presence/absence of pancreatic adenocarcinoma. Quantitative edge analysis can be promptly performed on select border fragments in MDCT images, providing a useful supporting tool for diagnostics and a possible starting point for machine learning recognition based on lower-dimensional feature space.

## 1. Introduction

Quantitative techniques are becoming increasingly used as valuable support for image analysis and computer-assisted diagnostics. By leveraging advanced mathematical algorithms and statistical methods, these techniques enable more precise and objective measurements of various anatomical structures and pathological findings in images. 

Multi-detector computed tomography (MDCT) quantitative analysis includes density measurements, volumetric analysis, perfusion CT, texture analysis, and radiomics. One of the key advantages of quantitative techniques in MDCT image analysis is their ability to provide quantitative data, such as the widely used measurements of lesion size, density, and perfusion parameters, which are crucial for accurate diagnosis and treatment planning. These techniques can also aid in detecting subtle changes in tissue characteristics that may not be readily apparent on visual inspection alone.

Furthermore, quantitative analysis can enhance the reproducibility and consistency of MDCT image interpretation by reducing the variability associated with subjective visual assessments. This is particularly important in computer-assisted diagnostics, where reliable and standardized measurements are essential for making informed clinical decisions.

Overall, the integration of quantitative techniques into MDCT image analysis represents a promising approach for improving the accuracy, efficiency, and reliability of diagnostic imaging in various medical applications.

Quantitative analysis involves the extraction of numerical data from images, enabling an objective analysis of various parameters that provide insights into disease processes, treatment responses, and patient outcomes, enhancing the accuracy of medical diagnosis and management. Quantitative imaging biomarkers are also promising in the development of new medical products, enabling an objective evaluation of the response to a specific therapy [1]. The subjective impressions of individual observers make consistent evaluations of radiology and pathology imaging difficult. Exploring methods to consistently translate complex visual observations into clearly defined algorithmic processes is a dynamic field of investigation with the potential to enhance clinical practice, advance investigative research, and enrich outcome studies [2]. An algorithmic procedure is fundamental from the perspective of applying deep-learning-based medical image analysis to computer-aided diagnosis (CAD), which can provide clinicians with decision support and improve diagnostic process accuracy and efficiency [3]. 

A recently introduced quantitative assessment technique is the analysis of the surface nodularity of parenchymal organs, used in medical imaging to assess the surface characteristics of anatomical structures, particularly in the context of multi-detector computed tomography and magnetic resonance imaging (MRI). This method involves analyzing the nodularity or irregularities on the surface of organs or tissues, which can provide valuable information for diagnostic purposes.

In the past, surface nodularity was assessed with visual inspection of the organ surface, a qualitative method. In the quantitative MDCT analysis of surface nodularity, advanced computational algorithms are employed to quantitatively evaluate the texture and morphology of the surfaces observed in CT images. This analysis can help identify subtle abnormalities, such as nodules, lesions, or other irregularities, that may be indicative of underlying pathology or disease.

One of the main advantages of MDCT surface nodularity analysis is its ability to provide objective and quantitative measurements of surface characteristics, which can supplement the traditional qualitative assessments made by radiologists. By quantifying surface irregularities, this technique may enhance the accuracy and reliability of diagnostic interpretations, particularly in cases where subtle changes may be difficult to detect visually.

The reliability of a quantitative evaluation of parenchymatous organ morphology via MDCT surface nodularity analysis has been widely demonstrated. The first software analyzing surface nodularity, successfully developed by Pickhardt et al., assessed liver surface nodularity (LSN) based on CT images and related it to the staging of hepatic fibrosis. This approach proved superior to visual methods in the evaluation of margin assessment and could become a noninvasive imaging biomarker of liver fibrosis [4]. 

Other dedicated studies have also demonstrated a correlation between LSN score and liver disease, supporting the possibility of the noninvasive staging of liver cirrhosis, thus suggesting possibly the broader applicability of surface analysis techniques for evaluating other organs and different diseases [4,5,6].

LSN quantification has then been demonstrated to be a biomarker for the detection and evaluation of cirrhosis [7] and could be used as a CT predictor of cirrhosis decompensation and death [8]. 

Hobeika et al. used LSN measurement as a practical tool in the preoperative evaluation and management of patients with HCC. The LSN score in the preoperative CT scan was associated with higher fibrosis and steatosis grade and was independently associated with severe complications and posthepatectomy liver failure [9,10]. 

The LSN score has also been used in the evaluation of liver fibrosis in non-alcohol-related liver disease (nonalcoholic fatty liver disease, NAFLD), a highly prominent liver disease in the world with a high economic burden [11].

The LSN score has been successfully used also to detect advanced liver fibrosis in CT scans in chronic viral hepatitis c and to identify portal hypertension [12,13]. 

Souhami et al. demonstrated that LSN has a similar performance as liver stiffness measurements (LSMs) using transient elastography (TE) for the detection of clinically significant portal hypertension in patients with compensated cirrhosis and hepatocellular carcinoma. In combination with other parameters, such as the score of the LSM spleen size to platelet ratio, portal hypertension could be identified in more than 75% of patients, enabling its diagnosis in centers where the hepatic venous pressure gradient is unavailable [14].

Overcoming the intrinsic limitations of software, even in a multi-institutional cohort of patients with chronic liver disease from HCV, the LSN score alone and in combination with the FIB-4 score exhibited strong diagnostic performance in detecting advanced fibrosis and cirrhosis [15].

Recently, LSN was defined as a predictor of liver failure in patients with colorectal cancer after chemotherapy [10].

After extensive liver surface nodularity analysis, the concept of quantitative surface nodularity evaluation was then applied to other abdominal organs, such as the kidneys and pancreas.

Regarding the kidneys, Ding et al. demonstrated a correlation between kidney surface nodularity and adverse vascular event risk in patients with arterial hypertension [16].

For the analysis of pancreatic margins, the surface nodularity score has been demonstrated to correlate both with glandular fatty infiltration recorded at pathology [17] and with exocrine functionality measured with laboratory tests [18].

In another study, pancreatic surface nodularity was also demonstrated to correlate with the postoperative onset of anastomotic fistula after pancreaticoduodenectomy—a major complication after pancreatic resections, significantly increasing morbidity and mortality [19].

Surface nodularity also seems promising in the detection of the focal pancreatic anomalies that determine alterations in the pancreatic surface. Pancreatic ductal adenocarcinoma is the most common primary malignancy of the pancreas (80% of malignant pancreatic neoplasms). It is a highly malignant tumor with a 5-year survival rate of 5%. It is an epithelial neoplasm with glandular differentiation: neoplastic glands are typically embedded in the desmoplastic stroma, and the tumor is surrounded by dense fibrosis, with changes in the morphological structure of the gland [20]. The neoplasm most commonly arises in the pancreatic head (60–70%) and causes symptoms such as jaundice, acute pancreatitis, pain that radiates to the back, and weight loss. 

The tumor is usually identified by imaging, focusing on the characteristics of the mass and of the adjacent parenchyma secondary to ductal obstruction [21]. 

Multiphasic MDCT is the imaging modality of choice for diagnosis and staging in patients with suspicion of pancreatic adenocarcinoma. The normal pancreas has lobulated margins surrounded by visceral fat. Pancreatic adenocarcinoma, instead, changes the structure of the pancreatic gland in terms of macroscopical features and vascularization. It is typically hypovascular to the surrounding pancreatic parenchyma and, because of the strong desmoplastic reaction, infiltrates the pancreatic and biliary ducts early [22]. 

So far, the reliable automated detection of healthy and neoplastic pancreas images has been a main challenge in radiological practice due to the relatively scarce amount of publicly available data, since radiologic data structures are not ready for artificial intelligence [23]. 

To address this issue, here, we introduce a specific image feature analysis, which focuses on the pancreatic surface, to differentiate normal pancreatic parenchyma from its most common focal lesion, adenocarcinoma. The differentiation of healthy parenchyma from adenocarcinoma through the analysis of glandular shape on nonenhanced MDCT images could provide a practical supporting tool for radiological practice and a convenient starting point for future machine learning analysis.

## 2. Materials and Methods

### 2.1. Patient Population 

The database of all patients with a pathological diagnosis of pancreatic adenocarcinoma over 24 months at the Pancreas Institute of the University of Verona was investigated. Informed consent for the utilization of clinical and radiologic data was provided by all patients (PAD-R registry, n1101CESC).

The inclusion criteria were as follows:

- Patients with adenocarcinoma of the tail of the pancreas;
- Diagnoses of adenocarcinoma confirmed at pathology of the resected specimen or at biopsy in case of unresectable neoplasms;
- Patients who underwent MDCT at our institute for the initial staging of the pancreatic neoplasm with images available for review on PACS.

The exclusion criteria were as follows:

- Adenocarcinoma of the head or body of the pancreas;
- No pathologically proven diagnosis of the adenocarcinoma;
- No MDCT images available for review.

### 2.2. Image Acquisition

All scans were performed on a 64-row MDCT scanner with a multiphasic acquisition that included a nonenhanced scan, a late arterial scan phase, and a portal venous scan phase. A weight-based amount (1.4 mL/kg) of high-concentration contrast agent (370 mgI/mL, Ultravist 370, BayerScheringPharma) was administered. The scans were read on PACS and dedicated workstations. 

### 2.3. Image Analysis–Pancreatic Edge Analysis Software

**Figure 1:**
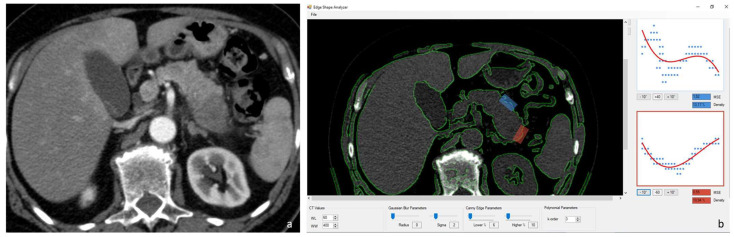
Postcontrast arterial MDCT axial image of adenocarcinoma of the tail of the pancreas (**a**). Screenshot of the GUI of the software used (**b**) with blue box positioned on normal parenchyma and the red box positioned on an adenocarcinoma of the tail of the pancreas. The graphic output of the results from the Canny edge detection and the subsequent average boundary line for both the boxes selected are visible.

Two radiologists, with 10 and 15 years of experience in abdominal imaging, analyzed the MDCT scans and selected through consensus two CT slices of the nonenhanced scan: one that depicted the tumor and one with normal pancreatic parenchyma. The images were annotated for further reference (Figure 1). “In-house” postprocessing software was used to quantify the amount of parenchymal surface nodularity on CT images as a continuous numerical value. The software used a Canny edge detector operator to identify a set of pixels as the pancreatic border defined as the average boundary line. Selecting a region of interest, the software provided a measure of the deviation of the analyzed pancreatic border from the smooth-fitted line. The root of these values is the “pancreatic margin score” (PMS) [18,19].

Pancreatic borders were sampled, drawing two boxes on CT slices with normal and tumoral parenchyma: the two boxes were kept constant in size in the different images and between different patients. The software automatically detected the pancreatic edge compared to the adjacent adipose tissues and provides as a graphic output the results from the Canny edge detection and the subsequent average boundary line together with all the relative results from the root mean square deviation calculation for an immediate comparison (Figure 1).

Overall, low PMS values indicate mild fluctuations around the average boundary line or, equivalently, a smooth edge, while high PMS values indicate important fluctuations around the average boundary line and an irregular margin. Larger PMS values are expected if the border is highly irregular (as in a healthy pancreatic gland), whereas smaller PMS values should be found if the actual border is well approximated by the fitted spline, as in pancreatic adenocarcinomas with important fibroblastic reactions.

The PMS values of normal pancreatic parenchyma and tumors were compared with paired *t*-tests. A receiver operating characteristic (ROC) curve analysis was conducted to evaluate the accuracy of PMS in the differential diagnosis. *p* < 0.05 was chosen to indicate significance. All statistical analyses were performed with commercially available software (GraphPad Prism version 7.01 for Windows, GraphPad Software, La Jolla, CA, USA, www.graphpad.com, accessed on 24 June 2024).

## 3. Results

**Figure 2:**
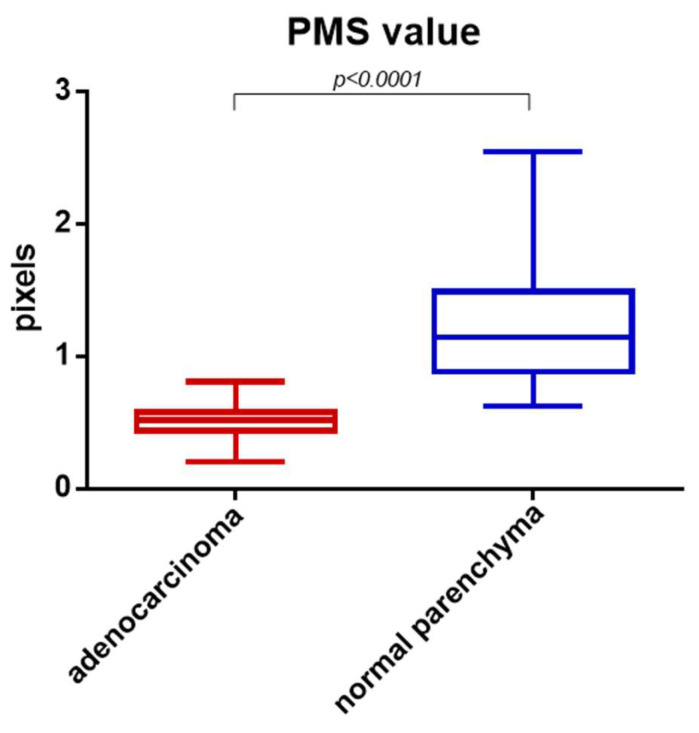
PMS values of cancerous and healthy pancreatic borders (whisker plot).

**Figure 3:**
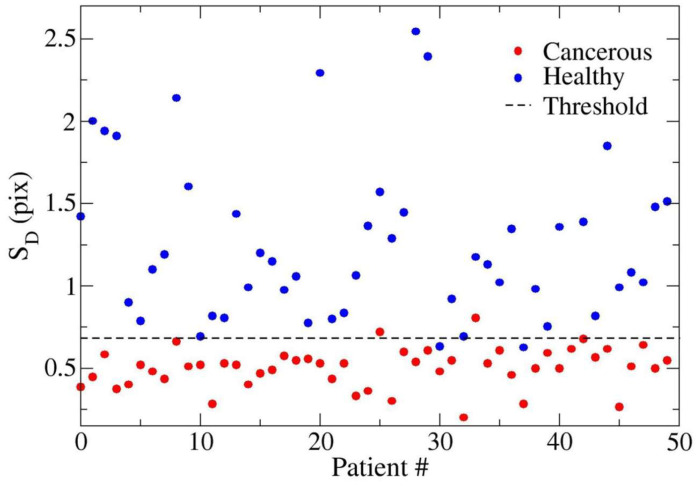
Visual plot of PMS data of adenocarcinoma and normal parenchyma and separation between cancerous (red) and healthy (blue) pancreas edge fragments.

**Figure 4:**
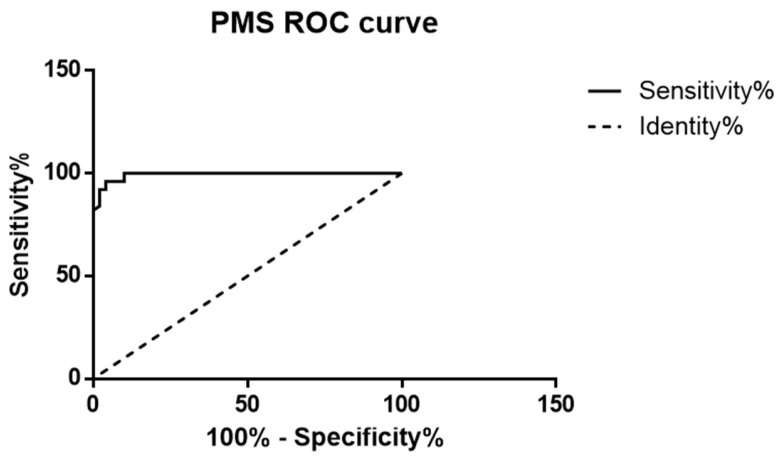
ROC analysis of PMS values of healthy and neoplastic pancreatic margins.

The final population included 50 patients (36 men, 14 women; mean age 63.7 years). In our series, cancerous border portions were systematically characterized by having significantly lower PMS values than healthy borders (0.5017 ± 0.1207 vs. 1.273 ± 0.4994; *p* < 0.0001) (Figure 2). The AUROC curve was 0.9926 for healthy normal parenchyma vs. pancreatic adenocarcinoma (95% CI 0.9825–1.003; *p* < 0.0001). In our series, the PMS value = 0.6855 pix acted as a threshold value, separating cancerous from healthy pancreatic edge segments (Figure 3) with 96% sensitivity and 96% specificity (Figure 4).

## 4. Discussion

The reliability of the quantitative evaluation of parenchymal organ morphology and its correlation with disease have already been demonstrated for the liver. It opens the way to noninvasive staging of cirrhosis, further suggesting broader applicability of surface analysis techniques [1,2,3,4,5,6]. Liver fibrosis has already been analyzed in alcoholic and non-alcohol-related liver diseases, such as in the post-chemotherapy and fatty liver.

Other organs are being investigated with the same technique, and promising results have been found in the pancreas that correlate pancreatic diffuse alterations (fatty infiltration), pancreatic exocrine function, and post-surgical complications (pancreatic fistula development).

The purpose of our study was to develop a specific image feature analysis method that focuses on the pancreatic surface to differentiate normal pancreatic glands from focal pancreatic lesions (in particular adenocarcinomas). 

Adenocarcinoma was chosen because it is the most common malignant pancreatic neoplasm and because of its smooth margins due to the important tumoral desmoplastic reaction. The healthy pancreas has irregular margins, making the lesion easily identifiable. The quantitative evaluation of the parenchymal surface irregularity (PMS) of healthy and cancerous pancreatic parenchyma was made by computing the root average quadratic SD of actual pancreatic edges from the corresponding average boundary line. As expected, the PMS values relative to cancerous pancreas samples were significantly lower than those in healthy organ margins (lesion borders are smoother than normal parenchyma). In our series, a threshold PMS value of 0.6855 could be used to differentiate between healthy and cancerous pancreas, demonstrating a 96% sensitivity and96% specificity in distinguishing pancreatic adenocarcinoma from normal pancreatic tissue. 

Due to their important desmoplastic reaction and early occlusion of the main pancreatic duct, adenocarcinomas of the pancreatic head and body cause early fibrotic alterations of the pancreatic parenchyma upstream from the lesions, making parenchymal margins smoother. To avoid this possible bias, we chose to analyze only lesions of the tail of the pancreas, which usually do not cause modifications of the normal parenchyma downstream in the head or body.

Radiomics is a rapidly evolving field in medical imaging that involves the extraction and analysis of numerous quantitative features from radiological images. While promising, it is also difficult to standardize, reproduce its results, and integrate into clinical practice due to its complexity. Pancreatic edge quantitative analysis has several advantages compared to other quantitative tests: it is rapid, easily reproducible, has high interobserver agreement, and can be performed on already-acquired CT scans. It also implies major computational advantages concerning straightforward full-image comparison. The limited amount of data publicly available for cancer diseases poses a main challenge for the naïve application of general-purpose image recognition techniques, given the relatively large dimensionality of MDCT image data. Quantitative edge analysis overcomes this issue by effectively reducing the scale of the problem, which may also provide a favorable starting point for focused machine learning applications. This should be considered as a pilot study that will be significantly improved based on the results of future studies.

This study has some limitations, namely, the relatively small series and the fact that we analyzed only patients with adenocarcinoma of the pancreas. 

Further studies are necessary to explore the use of this technique in other neoplastic and non-neoplastic diseases. Since this technique analyzes only the margins of the gland, it is necessary to evaluate if it can be used in the analysis of smaller tumors that are embedded within the parenchyma. Non-neoplastic applications have already been used to analyze fatty infiltration of the pancreatic gland and its correlation with exocrine function and postsurgery complications. Evaluating pancreatic fibrosis and its implications with this tool could be interesting. This was, however, an explorative study, performed on a small series of patients from a single center, examined on only one MDCT scanner. Further studies are warranted to increase the sample size and assess the reproducibility of the results across other image techniques (such as magnetic resonance), and between different CT scanners and other centers. 

A better standardization of the threshold values must be studied: we calculated the PMS values related to a virtual measure (pixels). Calculating PMS values associated with a physical measure (millimeters) could and should be explored to be able to compare the threshold values independent of the resolution of the images, making them reproducible.

The definition of multiple statistically relevant measures for border-segment corrugation indicates pancreas edge quantitative analysis as a promising feature assessment technique. 

Future directions in the field of organ surface nodularity quantification may include automatic nodule detection, the integration of nodularity data from different imaging techniques, and the creation of predictive models using radiomics techniques to correlate nodularity with clinical outcomes and treatment responses. Moreover, the evident connection between the present study and well-established diagnostic procedures indicates that the rational dimensional reduction in CT image analysis problems and possible feature-focused machine learning approaches could provide a promising pathway for the computer-assisted detection of rare cancers.

## Data Availability

The data presented in this study are available on request from the corresponding author. The data are not publicly available due to privacy restriction.
